# This time is different?—on the use of emergency measures during the corona pandemic

**DOI:** 10.1007/s10657-021-09706-5

**Published:** 2021-07-27

**Authors:** Christian Bjørnskov, Stefan Voigt

**Affiliations:** University of Hamburg, Hamburg, Germany

**Keywords:** COVID-19, Constitutional emergency provisions, State of emergency, Media freedom, Executive decrees, K40, Z13

## Abstract

The COVID-19 pandemic has not only caused millions to die and even more to lose their jobs, it has also prompted more governments to simultaneously declare a state of emergency than ever before enabling us to compare their decisions more directly. States of emergency usually imply the extension of executive powers that diminishes the powers of other branches of government, as well as to the civil liberties of individuals. Here, we analyze the use of emergency provisions during the first wave of the COVID-19 pandemic and find that it can be largely explained by drawing on political economy. It does, hence, not constitute an exception. We show that many governments have (mis-)used the pandemic as a pretext to curtail media freedom. We further show that executive decrees are considered as a substitute for states of emergency by many governments.

## Introduction

By May 10, 2020, 99 governments, equal to almost precisely half of all sovereign governments, had declared a state of emergency (SOE) due to COVID-19.^1^ The Italian government was the first to declare an SOE on January 31, 2020 and many other governments followed suit during March 2020. Such a wave of SOE declarations is completely novel. Between 1985 and 2014, the governments of at least 137 countries declared a state of emergency at least once (Bjørnskov & Voigt, 2018a based on updated data from Hafner-Burton et al., 2011). This number – spread over a period of 30 years - appears almost dwarfish compared to the current wave, which occurred in less than four months. As virtually all countries were affected by the SARS-CoV-2 virus at approximately the same time, governments were all fighting the virus, and their responses can be easily compared. This is akin to a natural experiment and constitutes a very rare setting enabling us to compare the determinants of declaring an SOE across virtually all countries in the world.

Alluding to both particular crises as well as governments’ responses to them, Reinhart and Rogoff (2009) entitled their book on centuries of financial crisis management *This Time Is Different.* Although during most crises, it is claimed that this time is really different, they showed that things weren’t so different after the financial crisis of 2008/9 and that history does seem to repeat itself. In this study, we ask a similar question: If hundreds of thousands are going to die and millions to lose their jobs, shouldn’t one assume that governments call an SOE because they want to save lives? Although emergency provisions have been misused time and again in the past, shouldn’t one expect that this time really is different?

Calling a state of emergency typically implies a shift in the balance of powers toward the executive to the detriment of the other government branches, but also to the detriment of citizens who enjoy fewer civil liberties. Sometimes, elections are postponed, parliaments are shut down, and if the courts are prevented from hearing cases, a judicial review of emergency measures applied by government is often close to impossible. There are already plenty of examples for the misuse of emergency provisions during the current pandemic. Casual observation seems to indicate that we have no reason to worry less this time around:

- Parliaments have been unable to meet in a number of countries due to the pandemic. The government of Mauritius, e.g., suspended its national assembly (Budoo, 2020), and the Thai government turned down the request of its parliament for an extraordinary session (Tonsakulrungruang & Leelapatana, 2020). Four days before declaring a state of emergency, the Serbian government prohibited gatherings of more than 100 people. Based on that decision, it argued that the parliament could not meet – with the consequence that the state of emergency was not confirmed by the Serbian parliament, which it is required to according to the Serbian constitution (Cavdarevic, 2020). Not to be controlled by parliament seems to be a desirable state of affairs for many governments.
- Court operations were suspended in many countries. Without doubt, this was often done to protect both parties involved in court cases as well as judges and court personnel. Yet, there are also cases in which the closing of courts simply means that government policies could not be challenged in court anymore. This has been the case in Bangladesh (Hoque, 2020) and Nigeria (Abdulrauf, 2020), among others. In Egypt, an amendment was passed that allows military courts to investigate any case discovered by a military officer. In other words: civilians can be tried in front of military courts again (Ellaboudy, 2020).
- In a number of countries, the military was brought in to enforce government measures. This was, e.g., the case in the theocracy of Iran (Alasti, 2020) as well as in democratic Malaysia and Denmark (Andersen & Bjørnager, 2020; Balasubramaniam, 2020). Domestic operations of the military always entail the danger that the military will remain an important player even after the state of emergency has been ended.
- Journalists have not only been targeted in repressive societies such as Belarus, where authorities on May 6 removed the official accreditation of Russia’s Channel One that had reported on the spread of the corona virus in the country (CPJ, 2020). In the United Kingdom, police threatened the journalist James Delingpole with fines if he reported on a protest against the government’s corona lockdown in London’s Hyde Park. The event was recorded by several people and went viral on Twitter. Similarly, the South African government has since mid-March prevented epidemiologists and other health experts from commenting on Covid-19 without coordinating with the National Institute for Communicable Diseases. In the Philippines, President Duterte refused to renew the license of the most popular Philippino television channel ABS-CBN (BBC, 2020).^2^

These are casual observations, and many more could be offered. Making judgments on a limited number of examples can be misleading – and even dangerous. This is why we are aiming at a more systematic analysis here. In this paper, we first inquire into the factors that provoked governments to declare an SOE. If declarations were clearly driven by the desire to save lives, things would be different. If, on the other hand, emergency declarations were motivated by political economy factors, things would not be so different after all. And indeed, we find that things are not so different. We then ask how governments have used the extra powers that they enjoy after declaring a state of emergency. It may still be premature to make a definite judgment regarding the effectiveness of lockdowns including restrictions to open shops or restaurants, to travel, to participate in religious ceremonies, to demonstrate or even to attend school or university and so on.^3^ Yet, one policy measure lends itself to judgment, namely how governments have behaved towards the media. This is why we ask to what degree media freedom has been upheld even after an SOE has been declared. We find that the protection of journalists – and free expression more generally – does not seem to be high on the agenda of many governments.

Very little is known about the factors determining the use of executive decrees in general. We hypothesize that some governments use them as an alternative to calling an SOE. We find that this is, indeed, the case with regard to reactions to SARS-CoV-2. We are not aware of any other studies having analyzed the relationship between SOEs and executive decrees. This constitutes, hence, another value-added of our study.

The rest of the paper is structured as follows: In Sect. 2, we briefly review the evidence on both the determinants of calling an SOE, as well as their effects. Section 3 spells out a number of hypotheses regarding the current pandemic as a possible cause for declaring an SOE. In Sect. 4, we describe our data and the estimation approach, and Sect. 5 contains the estimates. Section 6 concludes.

## Governments’ dismal record regarding emergency declarations

Today, 9 out of 10 constitutions contain constitutionalized emergency provisions, that is constitutions that spell out how and when an SOE can be declared and what additional competences the executive enjoys once it has been declared (Bjørnskov & Voigt, 2018a based on data described in Elkins et al., 2009). In the following, we refer to constitutionalized emergency provisions as “emergency constitutions” for simplicity although they are, of course, not a document different from a country’s constitution. Emergency constitutions such delineated differ widely regarding both formal content as well as specific detail. Whereas some constitutions only know a (unique) kind of emergency others differentiate between various kinds, often trying to rank the severity of the triggering event. In the empirical analysis we refrain from differentiating between different types of SOEs. These provisions have been invoked quite frequently: between 1985 and 2014, at least 137 countries reported at least one state of emergency declared on the level of the nation state (ibid. on the basis of updated data originally provided by Hafner-Burton et al., 2011).

To be able to ascertain the factors determining the employment of these provisions, we constructed an Index of Emergency Powers (INEP). The INEP consists of a Benefit INEP and a Cost INEP. The latter reflects how costly the constitution makes it for government to call an SOE and takes into consideration who has the power to declare an SOE (it is costly if the legislature or other bodies need to consent or have the power to declare it altogether), who has the power to approve an SOE (it is costly if the government needs the approval of other actors to declare a state of emergency) and the number of conditions named in the constitution as legitimate basis for declaring an SOE (the fewer conditions are named as justification for declaring a state of emergency, the more difficult it is to declare). The benefit components take into consideration whether, after having declared an SOE, the executive has the power to dissolve parliament, to suspend some basic rights, and the right to expropriate its citizens and censor the media. The INEP is coded as an additive index between 0 and 1 where 1 indicates complete (effectively dictatorial) powers to the executive. A high score on the cost component thus indicates low costs for the executive whereas a high score on the benefit components indicates a high level of benefits accruing to the executive during an emergency.^4^

How and in what sense do emergency constitutions impact on a government’s decision to declare a state of emergency? Comparing countries with and without an emergency constitution, Bjørnskov and Voigt (2018b) find that countries not having an emergency constitution are significantly more likely to declare a state of emergency than those having one (countries without an emergency constitution usually declare on the basis of statutory law). This is, hence, a first indication that constitutional constraints matter. Inquiring into the differences between emergency constitutions, Bjørnskov and Voigt (2018b) find that the less difficult it is for governments to declare an SOE based on the Cost INEP, the more likely governments are to do so.

What about the effects after having declared a state of emergency? Due to the very different characteristics of the events, it seems to make sense to distinguish between natural disasters on the one hand and political turmoil on the other. The rationale for declaring an SOE after natural disasters appears to be consistent with textbook public economics (cf. Barr, 2004). On the one hand, disasters such as earthquakes and volcanic eruptions are non-forecastable and, thus, not insurable and therefore arguably require some form of government intervention and support. On the other hand, epidemics are classic examples of situations with immediate negative externalities in the form of contagion, which may need some form of rapid government action to contain or slow its spreading. Calling an SOE can, therefore, be objectively necessary in order to effectively counter such negative externalities and related issues such as capacity problems in the health care system.

Yet, Bjørnskov and Voigt (2021a) report an unexpected result when analyzing natural disasters: Controlling for different disaster types (namely biological, geophysical, hydrological, and climatological) and the intensity of the disasters (by controlling for the number of people affected), they find that the higher the Benefit INEP, the *higher* the number of people killed as a consequences of a natural disaster. In other words: the more competences the constitution grants the executive under an SOE, the more people die, controlling for the intensity of the disaster. Moreover, the easier it is to call an SOE, the larger the negative effects on human rights. This seems to indicate that executives do not use the extra powers conferred on them during SOE to save lives but, rather, to their own advantage.

In their analysis of political turmoil, Bjørnskov and Voigt (2020) inquire into the effects of SOE declared subsequent to terrorist incidents. Once an SOE is declared, it generally leads to substantially higher repression levels by government. Yet, their main finding is that countries under an SOE are more, rather than less, likely to suffer from additional terror attacks, shedding doubts on the effectiveness of SOEs. That paper is based on a sample of countries that have a Western-style democratic constitution. The main results do, however, hold on the basis of a sample consisting predominantly of Northern African and Middle Eastern countries (idem, 2021b). This is noteworthy because this region is the most terror-prone in the world and most of these countries are autocratic. Despite being fundamentally different from natural disasters, SOEs declared during political turmoil appear equally counterproductive as in democracies.

Media freedom has been shown to make government action more transparent and accountable (Dreze & Sen, 1990). Unfortunately, it often suffers during and after emergencies. During a pandemic like SARS-CoV2 in which knowledge regarding the disease as well as effective means to fight it are scarce at first, it is particularly important as competing policy measures can be weighed and discussed widely only if the media is free to report on these. However, as noted by a number of studies, these mechanisms also imply that many governments and politicians are reluctant to respect media freedom and are interested in using emergency powers to curtail them (Clokie, 1947; Dragu, 2011; Hafner-Burton 2011; Bjørnskov & Voigt, 2021c).

In sum, it seems fair to be rather skeptical regarding the overall evidence of governments’ decision to declare an SOE. The decision to declare is decisively influenced by cost–benefit considerations of a government’s own utility, which may not reflect that of its citizens (cf. Buchanan & Tullock, 1962). When that is the case, governments typically do not resolve market failures and externalities, but actively impose negative externalities on the citizens as their welfare is not adequately internalized in the government’s utility function (Uslaner & Davis, 1975). It seems well justified to be at least as skeptical regarding how executives use their extra powers after having declared an SOE. More, rather than fewer, people die following a natural disaster and more, rather than fewer, terrorist incidents are observed after government responses to terrorist activities. In both types of disasters, SOE declarations are connected with substantially more government repression. This rather dismal record leads us to ask whether this time, i.e. with regard to COVID-19, is different.

## Hypotheses

Following directly from the dismal record of governments regarding their use of emergency powers just described, we hypothesize that this time is not different. Formulated differently: *The less difficult it is for government to declare an SOE, the more likely an SOE has been declared in relation to SARS-CoV-2*. And analogously: *the more benefits a government enjoys after having declared an SOE, the more likely an SOE has been declared in relation to SARS-CoV-2* (hypothesis 1a and 1b). If, however, this time really is different, then we would expect the state of public health with regard to COVID-19 to be the main determinant for declaring an SOE. These hypotheses are competing, and we run a horse race to see which hypothesis is more consistent with the data.

In the literature on the determinants of calling SOEs, executive decrees have played no role so far. We propose to change that here based on the conjecture that governments having at their disposal far-reaching executive decree powers have fewer reasons to resort to SOEs, *ceteris paribus*. Carey and Shugart (1998, p. 9) define decree power as “the authority of the executive to establish law in lieu of action by the assembly.” Power-maximizing executives that have been granted far-reaching decree powers by their country’s constitutions might, therefore, consider decrees and SOEs as substitutes. We conjecture that executives enjoying far-reaching decree powers will, indeed, be less likely to call an SOE. Argentine President Fernández, for example, did not declare a state of emergency during the pandemic but issued some 50 emergency decrees instead (Gargarella, 2020). The Indian government declared a tough nation-wide lockdown based on executive decrees only. A state of emergency was not declared – and parliament was not involved at any time (Bhatia, 2020). In both countries, states of emergency are extremely unpopular due to previous cases in which emergency powers have been blatantly misused, which might explain why governments relied upon their decree powers as a substitute.

We propose to control for three aspects, namely: the quality of political institutions, the level of economic development, and the capacity of the health system. The quality of political institutions can be ascertained in a variety of ways such as the degree to which the rule of law and press freedom are realized. In our context, the distinction between democratic and autocratic forms of government seems to be crucial. We assume that constitutions of democratic countries put more constraints on the executive than constitutions of autocratic countries. If the decision to declare an SOE is driven by the additional benefits associated with it, it follows directly that democratic governments have more incentives to declare an SOE.

The expected effect of controlling for the level of economic development is not entirely clear: during the pandemic, many governments have resorted to lockdowns, shutdowns, quarantine and the like. For people in poor countries without a full-blown welfare state, such measures can easily spell disaster. This would let us expect that poorer countries are less likely to declare an SOE. However, one may also argue that people in richer societies are more able to avoid risks of contagion that are endemic to poorer societies, and thus may not have a need for government-mandated lockdowns. Conversely, the effect of capacity in the health system is straightforward if government behavior is consistent with official justification of it. Many executives explicitly argued in March 2020 that a lockdown and other measures were necessary to protect hospitals and other parts of the health system from exceeding their capacity limits.

Governments do not enjoy getting criticized, a fact which holds for both democratic as well as autocratic governments. Disasters may therefore serve as a welcome pretext to curtail media freedom. Governments hope to get away with arguments such as `”curtailing media freedom only prevents rumors and false information to spread”, which could lead to additional uncertainty among the population. As a consequence of the pandemic, a number of countries have, indeed, passed legislation making the spreading of “fake news” a criminal offense although leaving the definition of “fake” an open question. We therefore hypothesize that *governments that declared an SOE also run more events against journalists*.

## Data and estimation approach

**Fig. 1:**
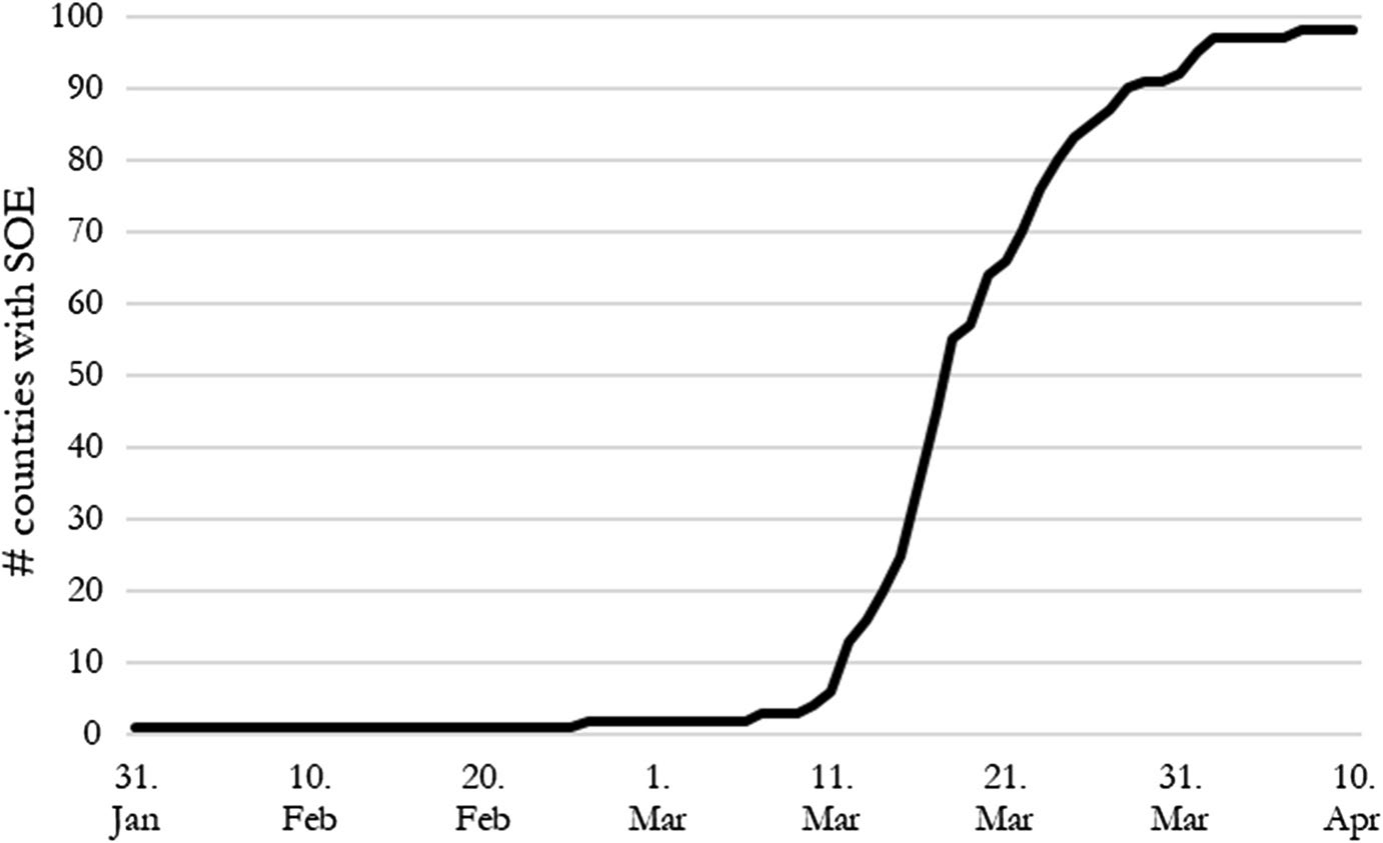
Emergency declarations

The central variable of this study is if and when countries declared an SOE. We coded this variable ourselves based on international news reports where we require at least two independent sources in order to code the onset of an SOE. We compared our list to one compiled by the Centre for Civil and Political Rights.^5^ Figure 1 documents the cumulative number of countries that had declared an SOE between January 31, 2020, and April 10, 2020. The first is Italy, which declared a SOE on January 31 with effect from the next day.

**Fig. 2:**
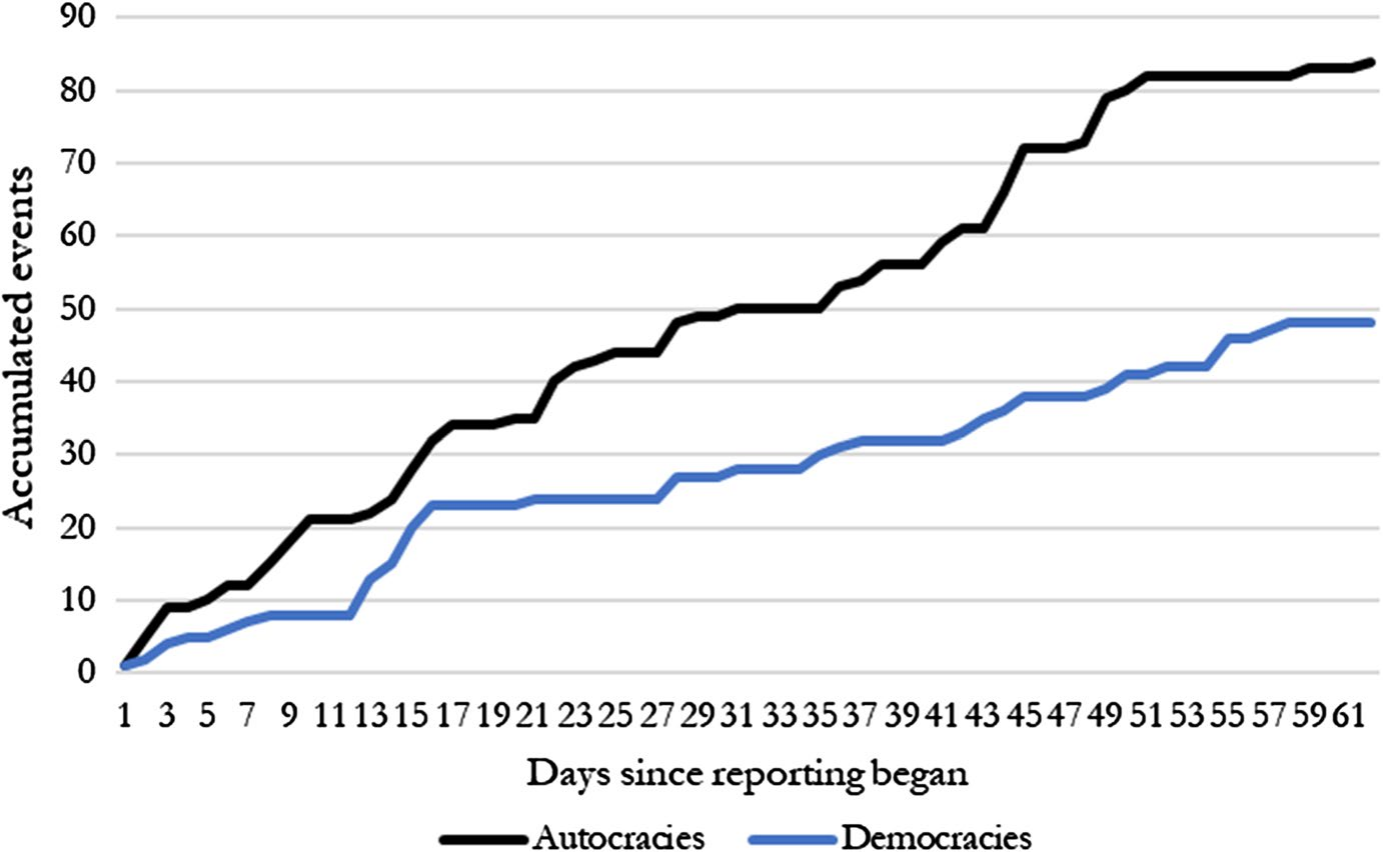
Repressive events against journalists and media

Next, we obtained events against journalists from the Eurozine/Index database, which is updated daily. The database is maintained at Eurozine, a network of European cultural journals that also publish an online magazine, and rests on daily reports on events against journalists from members of the network. The database covers a number of events against journalists, of which we only include the detention or arrest of journalists, journalists being prevented from reporting, new legislation that restricts media freedom and government U-turn on media policy (signals that government may with some probability no longer tolerate critical media), restrictions on social media, and crackdowns on so-called fake news. We supplement these data with a full confirmation of these events and our own media search for similar events since February 1, 2020, making sure that they were not related to other emergencies at the same time. The number of reported events against journalists is depicted in Fig. 2, in which we distinguish between democracies and autocracies.

We supplemented these data with information on a number of features. To code the power of governments to resort to executive decrees without having to declare an SOE, we created a new indicator, constitutionalized decree powers. It is based on a number of variables provided by the Comparative Constitutions Project (Elkins et al., 2009), coded such that the indicator can take on values between 0 and 2 with higher values indicating more government powers.^6^ A score of 0 means that no political actor has decree power while a score of 2 means that both the head of state and the head of government separately have full decree powers not constrained by parliament.

With respect to institutional and economic data, we employ the dichotomous democracy indicator from Bjørnskov and Rode (2020), the rule of law indicator from the Worldwide Governance Indicators (World Bank, 2021), the Reporters Without Borders (2020; RSF) index of violations of press freedom, the logarithm to real GDP per capita and population size from CIA (2020). We included these on the basis of the following expectations: democracies are expected to be more likely to declare an SOE because the power differential between normal and emergency times is larger than for autocracies. A high score in the rule of law indicates that governments have complied with abstract rules and that general rules are taken seriously in a high scoring country. This is why we expect governments with a high score to be less likely to declare an SOE. The media is free to discuss and criticize government behavior in countries that do well with regard to press freedom. Given that governments do not enjoy being criticized for limiting citizens’ civic freedoms, we expect governments in countries scoring well with regard to press freedom prior to the pandemic to be less likely to declare an SOE. From previous studies (e.g. Bjørnskov & Voigt, 2021a), we know that governments of more populous countries are not only more likely to declare an SOE but also to reduce civil liberties once an SOE has been declared. This is why we include it here too. In analyses of events against journalists, we effectively compare the number of events with the ‘normal’ situation in the country and with periods without an SOE.

In addition, we add the number of hospital beds per one thousand inhabitants as a measure of the capacity in the health system, thus providing a direct test of the official justification of most executive action in early 2020; the data are from the World Bank (2021). Finally, data on the number of people diagnosed with COVID-19, and the number of deaths associated with COVID-19 are from Our World In Data (Roser, Ritchie, Ortiz-Ospina and Hasell, 2020), which is also updated on a daily basis. Our health data covers January 1^st^ through May 10^th^, and we use the logarithm to the days since January 1^st^, the logarithm to the official number of infected, and a dichotomous indicator capturing whether any infected were observed on or before a given date.

In both estimates, we employ a logit estimator as both outcome variables are dichotomous; we employ random effects as a number of our main variables of interest are time-invariant within our relatively short time period. With SOE as the dependent variable, countries drop out of the sample once they have declared an SOE. While we only find 132 events against journalists, which may be considered a small number relative to the total number of observations, we do not believe it is sufficiently small to warrant the use of a rare events estimator or some form of rare events correction. Given that this type of correction might be needed, our estimators are likely to be conservative. Our unit of observation are country days. All variables are described in Appendix Table A1.

## Results

In this section, we first analyze the factors that lead governments to declare an SOE before turning to the question of whether declaring an SOE has any effects on the number of recorded events against journalists.

### Factors leading governments to declare

As described above, the general question is whether the declaration of an SOE is primarily driven by the state of public health with regard to COVID-19, or, alternatively, by the political attractiveness of calling an SOE. With regard to the state of public health, Table 1 shows that the (log) number of people affected make the declaration of an SOE more likely, but that neither the number of days passed since the first person infected was identified nor the question whether anybody was infected are significant determinants. We proxy the quality of the political system by the levels of democracy, the rule of law, and press freedom. It turns out that countries enjoying a high level of the rule of law as well as a high level of press freedom are less likely to declare an SOE, whereas neither the level of democracy nor the level of economic development are significant predictors for declaring an SOE. We also find no evidence that declaring an SOE is related to the capacity of the health system.

**Table 1:** Determinants for Declaring a SOE

	1	2	3
Democracy	−371	−8.442*	–
	(1.022)	(3.859)	
Rule of law	−4.232**	−4.336**	−4.934**
	(.892)	(.922)	(1.322)
RSF press freedom	−279**	−294**	−153
	(.058)	(.062)	(.085)
Log GDP per capita	.374	.122	1.413
	(.632)	(.678)	(1.053)
Log population size	−720*	−796*	−1.277**
	(.339)	(.369)	(.465)
Log hospital beds	.045	.065	−036
	(.585)	(.596)	(.742)
Log days since start	.306	.364	.205
	(1.064)	(1.119)	(1.162)
Log infected	1.066**	1.128**	1.367**
	(.238)	(.246)	(.314)
Any infected	1.789*	1.722	1.409
	(.867)	(.884)	(1.103)
Cost INEP	3.518	−7.927	6.059
	(3.312)	(5.578)	(3.775)
Benefit INEP	8.148**	7.714**	10.148**
	(1.962)	(2.529)	(2.815)
Cost at Democracy		15.455*	
		(6.747)	
Benefit at Democracy		2.094	
		(3.521)	
Observations	9665	9665	5896
Countries	158	158	95
Log likelihood	−371.563	−369.905	−257.545
Wald Chi sq	53.04	55.71	38.83

** (*) denote significance at *p* < .01 (*p* < .05). Estimates are obtained with a random effects logit estimator and include a constant term

According to the results reported in Table 1, the difficulty of declaring an SOE as measured by the Cost INEP is not a predictor for the observed wave of declarations. The additional powers that accrue to government once it has declared an SOE as indicated by the Benefit INEP are, however, highly significant. The interaction term in Column 2 even suggests that the effect of additional powers may be slightly stronger in democracies. As hypothesized above, the political attractiveness of gaining substantial discretionary power is, thus, not limited to autocratic governments. The last column of Table 1 refers to democracies only. By and large, democratic governments are driven by the same factors as autocratic ones. If anything, the amount of additional benefits a government enjoys once it has declared an SOE may play a more important role in democracies in comparison to autocracies such that democracies with particularly permissive emergency constitutions are indistinguishable from autocracies in emergencies.

Beyond the variables included in Table 1, we also asked if the generalized level of trust is associated with SOE declarations. It turns out that high trust democracies are significantly less likely to declare an SOE, but this result might be entirely driven by the Scandinavian countries. We did not include trust in the main models because the data are available for significantly fewer countries than those included in Table 1.^7^ We also asked whether extensive decree powers can be used by a government as an alternative to declaring a SOE. Governments – no matter whether democratic or autocratic – were significantly more likely to declare an SOE if decree powers were low and the benefits from declaring high than when decree powers were high and the benefits from declaring low. We summarize these findings in Table 2, where we show the probability that democracies (autocracies in parentheses) have declared an SOE, given that they are above or below the median of emergency powers and decree power.

**Table 2:** Probability of SOE Declaration Depending on Emergency as well as Decree Powers

		Decree index	
		Low	High
Benefit INEP	Low	0.79	0.41
		(0.18)	(0.45)
	High	0.79	0.60
		(0.57)	(0.20)

Numbers in parentheses pertain to autocracies

Taken together, these results are perfectly in line with previous findings: governments – no matter whether democratic or autocratic – are more likely to declare an SOE if this conveys more benefits to them and allows them to increase their competences to the detriment of both the other branches of government as well as citizens at large. In that sense, things have not been different this time. However, declaring an SOE does not automatically entail negative consequences for citizens. Therefore, we ask how governments have used their additional powers during the pandemic. We refrain from analyzing and evaluating the consequences of short-term measures such as quarantine, lockdown, or efforts to track and trace individuals, as it is truly challenging to assess the total consequences of such measures before the virus has run its full course. Rather, we are interested in trends that might very well have effects outlasting the current pandemic by, for example, changing policies and de facto institutions.

### Effects on media freedom

As a consequence of the pandemic, a number of countries have passed legislation making the spreading of “fake news” a criminal offense although leaving the definition of “fake” an open question. We therefore ask whether there is a significant association between events against journalists and SOEs.^8^

Our operational definition of “Events against journalists” includes new legislation to restrict media freedom, detention or arrest of journalists, journalists not allowed to report, and crackdown on “fake news”. Table 3 shows that fewer such events are expected in democracies, but are more likely in larger countries and after anyone had been infected. Conversely, we do not find any clear association with the stage of the pandemic (as measured by the number of days since December 31, 2019) or how many people were infected. Importantly, a government that has declared an SOE is no more likely to stage such events than governments that have not declared.

**Table 3:** Determinants of Events Against Journalists

	1	2	3
Democracy	−1.635**	–	.496
	(.379)		(1.218)
SOE	.405	.765	.422
	(.295)	(.546)	(.293)
Rule of law	−140	−307	−123
	(.222)	(.587)	(.222)
RSF press freedom	−000	.025	.004
	(.014)	(.041)	(.014)
Log GDP per capita	.227	.092	.236
	(.170)	(.506)	(.168)
Log population size	.680**	.787**	.664**
	(.105)	(.218)	(.108)
Log days since start	.423	.087	.403
	(.423)	(1.474)	(.422)
Log infected	−032	.012	−029
	(.055)	(.146)	(.056)
Any infected	1.661*	–	1.645*
	(.753)		(.753)
Cost INEP	−099	−3.405	.910
	(.743)	(1.881)	(1.313)
Benefit INEP	.210	.215	.466
	(.553)	(1.253)	(.643)
Cost with SOE			− 3.459
			(2.345)
Benefit with SOE			− 1.122
			(1.195)
Observations	11,344	6174	11,344
Countries	162	99	162
Log likelihood	−510.909	−145.349	− 509.223
Wald Chi sq	89.10	46.49	92.53

** (*) denote significance at *p* < .01 (*p* < .05). Estimates are obtained with a random effects logit estimator and include a constant term

It remains a possibility that passing fresh legislation curtailing media freedom is a precondition for observing events against journalists. Table 4 counts the number of events against journalists, taking into account whether any such legislation has been passed (both before and after possible events). According to the numbers reported in the table, it seems fairly irrelevant whether a country first introduced new legislation or not. Finally, a specific result is worth mentioning: when we only look at one particular category among the events against journalists, namely journalists arrested, and how often autocracies resort to such behavior, we find that 16% of those autocracies whose emergency provisions convey few benefits to the government have arrested journalists, compared to 52% of those autocracies conveying many benefits (p<.01). Clearly, the contents of emergency constitutions do seem to matter also in autocracies, while we find no clear differences among democracies. Note that the likelihood of arrests in democracies and autocracies with few emergency benefits does not differ significantly.

**Table 4:** Sequence of events against journalists

		De facto changes		
De jure changes		None	First	Second
	None	–	27 (13)	–
	First	8 (1)	–	5 (3)
	Second	–	7 (5)	–

Numbers in parentheses refer to democracies

In describing and evaluating government action during the pandemic, some other scholars have drawn conclusions somewhat different from ours. We take up two such views here and begin with Pozen and Scheppele (2020) who are concerned with what they refer to as “executive underreach.” They position this notion in direct opposition to executive overreach, in other words, the evaluation arrived at in this study.

Pozen and Scheppele (2020) are concerned with (heads of) governments who are not sufficiently active in protecting their populations against the pandemic and explicitly refer to Brazilian president Bolsonaro and U.S. president Trump. In principle, such an evaluation is completely compatible with ours: that there are many governments that overreach does, of course, not exclude some governments that underreach. More to the point, underreach is not necessarily equivalent with the decision not to declare a state of emergency. We insist that there exist many potentially effective policy options below the declaration of a state of emergency.

In a paper entitled “The Bound Executive: Emergency Powers during the Pandemic”, Ginsburg and Versteeg (2021) argue that legislatures, courts, and subnational government units have played an important role in constraining national executives and that the claim of executive overreach might, therefore, be overblown. It is definitely true that in many countries, these actors have behaved as veto players who have monitored the executive and also constrained it somehow. We are less optimistic in interpreting the overall evidence, however. The simple fact that in many countries, the respective top courts have dealt with dozens of suits in which the executive was held to have overstepped its competences can not only be interpreted as evidence in favor of the constraining role of the judiciary, but also in the frequent attempts of the executive to broaden its competences to the detriment of citizens’ freedoms.

## Conclusions and outlook

Summing up, it seems safe to conclude that this time is not different. As under previous natural disasters, democratic and autocratic governments alike have behaved like power-maximizers during at least the first wave of the corona pandemic. We find that the discretionary power they gain during emergencies is the main determinant of whether they declared a state of emergency, while the severity of the epidemic is irrelevant. To our knowledge, this is the first paper that asks whether executives perceive executive decrees as substitutes of states of emergency and use them accordingly. With regard to SARS-CoV-2, we show that this is, indeed, the case. Future research should ask whether this insight also holds with regard to states of emergency not related to SARS-CoV-2.

We also observe that those executives that have declared a state of emergency as a consequence of the pandemic may be likely to misuse these powers against journalists and the media. The danger, as under previous disasters, is that some of the measures now implemented are likely to outlast the current pandemic and weaken the rule of law and democracies for many years to come. In fact, in many countries the ultimate victim of the corona virus may be the separation of powers and freedom of expression.

What implications follow from these results? This question is pressing as Lührmann and Rooney (2021) observe that the declaration of an SOE often initiates the process of democratic backsliding. Getting rid of emergency constitutions entirely is no adequate answer. As observed by Bjørnskov and Voigt (2018b), countries without an emergency constitution are more likely to declare an SOE than countries having incorporated emergency provisions into their constitutions, c.p. More generally speaking, incorporating emergency provisions into the constitution makes government behavior more predictable, independently of the reduced likelihood of declaring.

To reduce the likelihood that the contemporaneous use of emergency provisions will have long-term repercussions on the separation of powers and the rule of law more generally, it seems to make sense to include a number of provisions into emergency constitutions. First, states of emergency should only be allowed for a limited time clearly spelled out in the provisions. Second, any legislation passed under a state of emergency should automatically cease to be valid with the end of the emergency. To keep it beyond the emergency phase, legislation should be subject to broad public discussion and pass the conventional parliamentary readings again. Both these insights were realized in the Roman Republic more than two millennia ago but appear to have been forgotten in the meantime. Third, the judiciary could be allocated the power of ex post-judicial review. On the one hand, experience with such provisions are not entirely encouraging. On the other, apex courts have increasingly demonstrated their willingness to constrain governments in their behavior, so allocating them with the competence to review government behavior promises to make executives more rule abiding in their implementation of a SOE. Regardless of one’s ideological position and constitutional preferences, the dramatic use of SOEs during the SARS-CoV-2 should force all political actors as well as the media and academics to reassess the use of emergency powers.

## Appendix

We compose our index of decree powers based on variables from the Comparative Constitutions Projekt (Elkins et al., 2009). We first enter whether the head of government has executive power or not, coded as 0 or 1; when the constitutional status is uncertain, we code it as 0.5. We next construct a categorical variable capturing whether a decree is effective immediately upon announcement, if it is effective following a constitutionally mandated period during where an approving body can potentially repeal it, or if it is only effective after being approved by some other body. These two indices are averaged and scaled between 0 and 1. We construct exactly similar indicators for the head of state, and add the two indicators such that the maximum value is 2 – when both the head of state and head of government have unrestricted decree powers – and the minimum is 0 when the executive have no decree power.

See Table

**Table 5:** Descriptive statistics

	Mean	Standard deviation	Observations
SOE	.277	.447	13,527
Event against journalists	.009	.096	11,371
Democracy	.642	.479	13,527
Rule of law	.169	1.026	13,527
RSF press freedom	33.108	15.270	13,527
Log GDP per capita	9.687	1.128	13,527
Log population size	16.394	1.752	13,527
Log hospital beds	.786	.890	13,403
Log days since start	4.186	.728	13,527
Log infected	3.872	3.380	13,490
Any infected	.748	.434	13,526
Cost INEP	.525	2.094	13,527
Benefit INEP	.393	.223	13,527

^5^.

## Footnotes

1 After May 10, no more COVID-19 related states of emergency referring to the first wave of the pandemic were recorded. Indeed, the last nation to call a state of emergency owing to the corona pandemic was Japan on April 7. We cap the period we are looking at in early May so not as to deal with the end of emergencies that came about in a few countries for very different reasons. Most pertinently, the El Salvadorian Supreme Court revoked the government’s continuation of its SOE as it would include unconstitutional transfers of specific powers to the executive.

2 The *Committee to Protect Journalists* has since March kept a list of infractions of press freedom and violence against journalists. Approximately 20 percent of their cases are from democratic countries.

3 Although first studies are, of course, already available. The data-driven studies include Atkeson et al. (2020), Bendavid et al. (2021), Bjørnskov (2021) and Chaudhry et al. (2020) who all find no evidence of lockdown effectiveness.

4 The INEP was first introduced in Bjørnskov and Voigt (2018a) which contains more details regarding its construction and coding.

5 Available at https://datastudio.google.com/u/0/reporting/1sHT8quopdfavCvSDk7t-zvqKIS0Ljiu0/page/dHMKB (last assessed on Oct. 19, 2020). There are four countries included in our list but not in theirs. These are Antigua, Kosovo, Sao Tomé and St. Lucia. After doublechecking, we decided to keep these four countries in. On the other hand, their list includes a number of countries that we do not include. These are countries that had been in a state of emergency even before the outbreak of SARS-CoV-2 such as Egypt and Tunisia. Since we are interested in states of emergency that are due to the pandemic, we decided not to include them into our dataset.

6 Details regarding the components of the new indicator and its construction are contained in the appendix.

7 These results are available upon request. The measure of social trust used in them follows the standard in the literature by using the dichotomous question of whether people believe that most people can be trusted. The data derive from the World Values Survey as well as the Barometer surveys.

8 There are various measures for media freedom. Unfortunately, none of them are available for 2020 yet which is why we resort to the number of events directed against journalists.
